# Urinary incontinence among pregnant women, following antenatal care at University of Gondar Hospital, North West Ethiopia

**DOI:** 10.1186/s12884-016-1126-2

**Published:** 2016-10-28

**Authors:** Abey Bekele, Mulat Adefris, Senait Demeke

**Affiliations:** 1 Department of Physiotherapy, School of Medicine, University of Gondar, Gondar, Ethiopia; 2 Department of Obstetrics and Gynecology, University of Gondar, Gondar, Ethiopia

**Keywords:** Antenatal care, Ethiopia, Pregnant women, Prevalence, Urinary incontinence

## Abstract

**Background:**

Urinary incontinence is defined as a complaint of any involuntary leakage of urine. During pregnancy, the prevalence of urinary incontinence ranges from 32 to 64 %. Different factors like demographic factors, obstetric factors, and other external factors affect urinary incontinence. In Ethiopia, there is no study conducted so far on the magnitude of urinary incontinence and factors associated among pregnant women. The objective of this study was to determine the prevalence of urinary incontinence and associated factors among pregnant women following antenatal care at the University of Gondar Hospital.

**Methods:**

Institution based cross- sectional study was conducted among 422 pregnant women following antenatal care at the University of Gondar Hospital. Data was collected using a structured questionnaire and analyzed using SPSS version 20. Descriptive, bivariate, and multivariate analyses were performed. The results were considered significant at *p*-value < 0.05.

**Result:**

The overall prevalence of urinary incontinence among the participants was 11.4 % [48]. After adjustment episiotomy, constipation, obese women, chronic cough/sneezing, asthma/allergies/sinusitis was associated with urinary incontinence.

**Conclusions:**

In this study, a lower prevalence was found than that of previous studies. There was a significant association of urinary incontinence with a previous history of episiotomy, constipation, maternal BMI, and respiratory problems.

## Background

Urinary incontinence [UI] is defined as a complaint of any involuntary leakage of urine [1]. It has been reported to affect 5 - 69 % of women [2]. The prevalence of UI is higher in specific subgroups, such as pregnant women, where it is estimated to occur in 32–64 %. Stress and mixed incontinence contribute 59 % and 40 % of the cases respectively [3–7].

Pregnancy is a well-known risk factor for UI, this is due to the physiologic and anatomic changes, especially in the third trimester, that can result in weak pelvic floor muscles (PFM) [3, 8]. Other risk factors could be the age of the mother, parity, previous delivery, body mass index [BMI], and UI before pregnancy [3, 5, 9–11].

The prevalence of UI during pregnancy in Europe has been reported to be 26–71 % [4–6]. Similarly, in north and South America estimated to be 43–63 % [3, 4, 12].

Since women in low-income countries are vulnerable to the risk factors like being multiparous, lack of adequate health infrastructures, lack of intervention for UI and low attitude towards it [3, 6, 9, 10, 12], it is possible that UI being common and affect the daily life of pregnant women more severely than suggested by reports from high-income setting. However, there has been few studies conducted [13, 14].

In Ethiopian women being ashamed, embarrassed and fear of being discriminated led to hiding their problem [15]. Despite, other factors like high fertility rate, a difference in lifestyle, environmental and genetic factors, a different health care system especially antenatal Care [ANC] and delivery care affecting the prevalence of UI there was no study conducted so far.

The aim of this study was to determine the prevalence and associated factors of UI among pregnant women following Antenatal Care at the University of Gondar Hospital.

## Methods

An institution based cross-sectional study was conducted from February to June 2014 on 422 pregnant women who were following antenatal care [ANC] clinic of the University Of Gondar Hospital [UOGH]. In the year, 1954 the hospital was established. It is located 741 km away from Addis Ababa, situated in Gondar town, North West Ethiopia. The hospital is a referral for four district hospitals and serves more than 5 million people in the region.

A total of 422 pregnant women who were following ANC at UOGH during the study period were included in the study. The study samples were selected using Systematic sampling from the total pregnant women who had follow-up at the ANC by using *K* = 2. Whereas pregnant women who were severely sick, diagnosed with kidney or urethral infection and women with contra-indication of vaginal palpation was excluded from the study.

The data were collected using a structured questionnaire adapted from similar studies and amendments were done to fit it with the local context [6, 7, 16]. The questionnaire was translated from English to Amharic. Information on socio-demographic characteristics, obstetric factors, and other factors were collected. A pretest was done on 10 pregnant women who have been following ANC at UOGH, then modification was made on the definition of stress, urge, and mixed UI to enhance the consistency of understanding the questions by the respondents and as well as by the data collector.

‘Urinary incontinence’ was defined as the complaint of any involuntary leakage of urine at least once during their current pregnancy,’stress urinary incontinence’ as the complaint of involuntary leakage on effort or exertion or on sneezing or coughing,’urge urinary incontinence’ as the complaint of involuntary leakage accompanied by or immediately preceded by urgency [strong inner drive to urinate].’ Mixed urinary incontinence’ is defined as the complaint of involuntary leakage in association with urgency and with exertion effort, sneezing or coughing.

Two trained midwives with Bachelor of Science degree (BSC) and working at the ANC follow-up clinic collected the data and performed the pelvic examination. They trained for 3 days by the primary investigator on the objective of the study, data collection procedures, and assessment of pelvic floor muscle [PFM] strength using digital palpation method.

PFM strength was examined by placing the participants lying on their back with knees bent and feet flat on the bed [crook lying]. The hips were abducted and covered with a sheet and they were instructed about the exact way to perform PFM contractions. A single examiner evaluated the participants, by using the bi-digital [index and middle finger] vaginal palpation method; this was conducted in a separate room.

The PFM strength was graded using the modified Oxford scale which is a validated tool for the measurement of PFM strength on bi-digital vaginal palpation [16, 17].’ Grade 0’ is no discernible PFM contraction,’Grade 1’ is a flicker or pulsing, ‘Grade 2’ is weak contraction,’Grade 3’ is moderate contraction, ‘Grade 4’ is good PFM contraction, and ‘Grade 5’ is a strong contraction of the PFM. Grade 0–3 are said to be weak muscle strength while grade 4–5 strong muscle strength.

Height was measured using stadiometer and weight using a calibrated weighing machine. Body mass index was calculated and BMI <20 is taken as underweight, ‘Normal’ as [20–29.4], ‘Overweight’ as [25–29.9] and ‘Obese’ as BMI > 30 [18].

Constipation was evaluated by the frequency as ‘never’ being experiencing constipation less once, ‘rarely’ being experiencing constipation one or several times a month, ‘sometimes’ being experiencing constipation one or several times a week and ‘often’ being experiencing constipation everyday during pregnancy.

The data collectors were supervised during the data collection period and the data was checked for its completeness, accuracy, and clarity on a regular basis by the primary investigator during the data collection period. The data was entered and analyzed by using SPSS version 20. Collinearity test, descriptive, bivariate and multivariate analysis was performed. Factors with *p*-value < 0.2 % in the bivariate model were further analyzed using multivariate model.

Multiple logistic regressions were used to control for possible confounders and to examine the association between different independent variables with the outcome variable. The results were considered significant at *p*-value < 0.05.

### Ethical considerations

Ethical clearance was obtained from School of medicine, ethical review committee of the University of Gondar. After the purpose of the study was explained, written consent was obtained from each voluntary participant. During the data collection and examination, a separate room was used. Participants were allowed to quit at any time of the data collection if felt uncomfortable. They have also informed their participation in the study has no effect on the care that they receive. All the information was kept confidential at any stage of the study. All participants with UI were referred to the department of physiotherapy for further management.

## Results

A total of 456 women were approached where 422 participated in the study with 92.5 % response rate. The mean age of participants was 26 years [range 16–40 years]. One hundred forty [33.2 %] participants attended secondary school and 254 [60.2 %] were a housewife. Urban residents were 345 [81.8 %] and 378 [89.6 %] were orthodox Christians [Table 1].

**Table 1:** Socio-demographic characteristics of pregnant women following ANC at University of Gondar Hospital, March 2014, Gondar, Ethiopia [*N* = 422]

Characteristics		Distribution
		*N* [%]
Age	16–24	143 [33.9]
	25–29	172 [40.8]
	30–34	60 [14.2]
	35–40	47 [11.1]
Education status	Unable to read and write	62 [14.7]
	Read and write only	8 [1.9]
	Grade 1–8	76 [18]
	Grade 9–12	140 [33.2]
	College level	136 [32.2]
Occupation	Housewife	254 [60.2]
	Teachers	40 [9.5]
	Nurses	2 [0.5]
	Office workers	67 [15.9]
	Others	59 [14.0]
Marital status	Married	407 [96.4]
	Single	12 [2.8]
	Divorced	3 [0.5]
Residence	Urban	345 [81.8]
	Rural	77 [18.2]
Gestational age	1–3 month	41 [9.7]
	4–6 month	101 [23.9]
	7–9 month	252 [59.7]
	>9 month	28 [6.6]

The majority of women, 254 [60.2 %] had normal BMI during the current pregnancy. Two hundred thirty-six [55.9 %] of them had never experienced constipation. The weakness of the PFM muscles was found in 49 [11.6 %] women [Table 2].

**Table 2:** Clinical characteristics of pregnant women following ANC at University of Gondar Hospital, March 2014, Gondar, Ethiopia [*N* = 422]

Characteristics		Distribution
		*N* [%]
Parity	0	132 [31.3]
	1–3	243 [57.6]
	≥4	47 [11.1]
Prior abortion	None	330 [78.2]
	Once	73 [17.3]
	Twice or more	19 [4.5]
Mode of Previous delivery	None	161 [38.2]
	Vaginal delivery	36 [8.5]
	Instrumental delivery	11 [2.6]
	Caesarean section	214 [50.7]
	Episiotomy	45 [10.66]
Constipation	Never	236 [55.9]
	Rarely	107 [25.4]
	Sometimes	48 [11.3]
	Often	31 [7.3]
Respiratory problems	Chronic cough/sneezing	36 [8.5]
	Asthma/Allergies/ sinusitis	35 [8.3]
	None	351 [83.2]

One hundred thirty-two [31.3 %] women were primigravid. From the total women 73 [17.3 %] had a Prior abortion at least once. Two hundred fourteen [50.7 %] women had caesarean section and 45 (10.66 %) had episiotomy during their previous delivery and 28 [6.6 %] had experienced UI during their earlier pregnancy [Table 2].

The overall prevalence of UI among the participants was 11.4 % [48]. Of all the respondents with UI 22 [45.8 %] of them reported that they have UI during the second trimester and 20 [41.6 %] participants had incontinence once or several times a week. From the total women with UI 37 [77.1 %] had weak pelvic floor muscles and 25 [52.1 %] had UI during their previous pregnancy [Table 3].

**Table 3:** Characterstic of Urinary incontinence among pregnant women at the University of Gondar hospital, March 2014, Gondar, Ethiopia (*N* = 422)

Characteristics		Urinary incontinence	
		*Yes (*%*)*	*No (*%*)*
Age	16–24	14 (29.2)	129 (34.5)
	25–29	20 (41.7)	152 (40.6)
	30–34	6 (12.5)	54 (14.4)
	35–40	8 (16.7)	39 (10.4)
Gestational age	1–3 month	2 (4.2)	39 (10.4)
	4–6 month	14 (29.2)	87 (23.3)
	7–9 month	28 (58.3)	244 (59.9)
	>9 month	4 (8.3)	24 (6.4)
Pelvic floor muscle strength	Strong	11 (22.9)	362 (96.8)
	Weak	37 (77.1)	12 (3.2)
History of UI during previous pregnancy	Yes	25 (52.1)	371 (99.2)
	No	23 (47.1)	3 (0.8)
History of UI(life time)	Yes	25 (52.1)	363 (91.1)
	No	23 (47.9)	11 (2.9)
Frequency Of UI	Less than once a month	4 (8.3)	-
	One or several times a month	8 (16.6)	-
	One or several times a week	20 (41.6)	-
	Every day	16 (33.3)	-

A significant association of UI was found with having episiotomy [AOR 4; 95 % CI 1.2–12.57], with having constipation sometimes [AOR 7; 95 % CI 2.5–19.9] and having constipation often [AOR 12; 95 % CI 3.6–40.5] [Table 4].

**Table 4:** Association between urinary Incontinence and selected variables among pregnant women at university of Gondar hospital, March 2014, Gondar, Ethiopia [*N* = 422]

Characteristics		UI		Crude OR [95 % C]	Adjusted OR [95 % CI]	*P*
		yes	No			
History of surgery	None	31	311	1	1	
	Episiotomy	9	36	2.5 [1.1–5.68]*	3.9 [1.2–12.57]**	0.023
	Gynecological surgery	6	16	3.7 [1.37–10.3]*	19.4 [3.2–117]**	0.001
	Abdominal surgery	2	11	1.82 [0.38–8.6]	0.54 [0.4–7.28]	0.644
Constipation	Never	14	222	1	1	
	Rarely	5	102	0.78 [0.232.216]	0.70 [0.20–2.49]	0.592
	Sometimes	16	32	7.9 [3.5–17.78]*	7.19 [2.5–19.9]**	<0.001
	Often	13	18	11.45 [4.7–28]*	12 [3.6–40.5]**	<0.001
Parity	0	14	118	1	1	
	1	9	117	0.64 [0.27–1.55]	0.43 [0.12–1.56]	0.201
	2	11	71	1.3 [.56–3.033]	0.66 [0.16–2.72]	0.564
	3	9	26	2.9 [1.14–7.46]	1.61 [0.38–6.88]	0.512
	≥4	5	374	1.003 [.34–2.95]	0.6 [0.12–2.91]	0.527
Respiratory problems	None	24	327	1	1	
	Chronic cough/sneezing	10	26	5.24 [2.2–12.1]*	4.05 [1.5–10.5]**	0.005
	Asthma/Allergies/sinusitis	14	21	7.6 [3.0–19.15]*	10.6 [3.4–33.2]**	<0.001
Mode of pervious delivery	None	17	144	1	1	
	vaginal	4	32	1.05 [0.33–3.36]	0.6 [0.14–2.51]	0.485
	instrumental	1	10	0.85 [0.11–0.70]	0.4 [0.4–4.41]	0.566
	Caesarean	26	188	1.17 [0.61–2.24]	1.23 [0.14–10.9]	0.848

*significant *p* < 0.2 1 = reference **significant *p* < 0.05

## Discussion

This facility-based study of urinary incontinence among pregnant women showed the prevalence of UI was 48 [11.4 %]. This was lower compared with previously conducted study in Europe, where the prevalence of UI ranging from 26–71 % [4–6] and in north and south America ranges from 43–63 %[3, 19]. However the finding of this study was found to be in line with that of the prevalence in South Africa reported to be 12 % [20].

The difference in the prevalence was explained in the Norwegian study that, the prevalence of UI was found to be lower in pregnant women with African origin when compared with Europe/North American origins [7]. This finding is also supported by a study conducted in California; they found that black women are at less risk of having a UI that Hispanic and white women [21].

Another explanation is the difference in the age distribution, where the current subjects have a narrower range than others, e.g. The Brazilian study ranges 14–44 [3], and a younger population compared to the others. On the baseline characteristics, this study has a non-smoking population and with normal BMI during pregnancy. In previous studies, like the Brazilian study, smokers and obese subjects were included in the study [3, 6, 22] which were found to be associated with UI during pregnancy.

The other possible reason for the lower prevalence rate can be under-reporting. In this study 2.84 % of the subjects with a weak PFM, which is a significant predictor of UI, on the objective assessment, did not report on having UI during the subjective evaluation. A study in Ethiopia showed that reporting UI and seeking medical attention is very limited [15]. Secrecy of UI is raised upon the feeling of shame, embarrassment, and fear of discrimination [15]. The other reason might be that urine leakage is considered as normal during pregnancy and may not be differentiated from normal vaginal discharge that occurs during pregnancy [2].

In this study, the proportion of stress UI was 58 %, mixed UI 24.5 % and urgency UI 12.5 %. This finding is similar to studies done in Australian, Turkish, Taiwanese, and Chinese pregnant women [5, 6, 21]. The previous history of surgery was found to be significantly associated with current UI during pregnancy. Women who had gynecological surgery and episiotomy were 19.4 and 4 times more likely to have UI during their pregnancy respectively. This was supported by a Danish study reporting a significant association of UI with gynecological surgery [17].

Another significant association was also found among women who experienced constipation during the current pregnancy. In this study, women who often experienced, constipation is 12 times more likely to have UI, whereas women who sometimes experience constipation are 7 times more likely to have a UI. This might be due to overloading during defecation as a result constipation and resulted in damaged PFM that contribute to UI.

In this study, a significant association was found between UI and having a respiratory problem during pregnancy. Pregnant Women with asthma/allergies/sinusitis and with chronic cough/sneezing were 10 and 4 times likely to develop UI respectively when compared with pregnant women without respiratory problems. Respiratory problems were not studied in many of the studies even though the Turkish study also found a significant association of respiratory problem during pregnancy with UI [5]. Respiratory problems like a chronic cough and sneezing will increase intra-abdominal pressure, increasing the pressure on the PFM along with the pregnancy itself. This will progressively loosen the muscles resulting in urinary incontinence.

Some of the factors like smoking, maternal age, gestational age; prior miscarriage and parity were not associated with UI in the current study. This difference can be due to the difference in health care systems, population characteristics and the relatively smaller sample size in this study.

The Lack of use of biofeedback as evaluation of PFM and the reliance on self-report of UI rather than objective assessments could be some of the limitations of this study and which might have led to under-reporting of UI.

## Conclusions

In this study the prevalence of UI during current pregnancy was found to be lower compared to previous studies conducted. The previous history of surgery, constipation, obesity and respiratory problems were found to be significantly associated with UI during pregnancy. Further research is required on a larger sample size, involving objective examination and better assessment equipment of pelvic floor strength [e.g. Perineometer or biofeedback].
